# Evaluating a nurse-led survivorship care package (SurvivorCare) for bowel cancer survivors: study protocol for a randomized controlled trial

**DOI:** 10.1186/1745-6215-14-260

**Published:** 2013-08-19

**Authors:** Michael Jefford, Sanchia Aranda, Karla Gough, Kerryann Lotfi-Jam, Phyllis Butow, Mei Krishnasamy, Jane Young, Jo Phipps-Nelson, Lahiru Russell, Dorothy King, Penelope Schofield

**Affiliations:** 1 Department of Cancer Experiences Research, Peter MacCallum Cancer Centre, East Melbourne, VIC 3002, Australia; 2 Sir Peter MacCallum Department of Oncology, The University of Melbourne, Parkville, VIC 3010, Australia; 3 Faculty of Medicine, Dentistry and Health Services, University of Melbourne, Parkville, VIC 3010, Australia; 4 Cancer Institute NSW, PO Box 41, Alexandria, NSW 1435, Australia; 5 Department of Nursing, School of Health Sciences, The University of Melbourne, Parkville, VIC 3010, Australia; 6 Surgical Outcomes Research Centre, University of Sydney and Sydney South West Area Health Service, Missenden Rd, PO Box M157, Sydney, NSW 2050, Australia; 7 Centre for Medical Psychology and Evidence-based Decision Making (CeMPED), School of Psychology, University of Sydney, A18, Sydney, NSW 2006, Australia; 8 School of Public Health, University of Sydney, A27, Sydney, NSW 2006, Australia; 9 Surgical Outcomes Research Centre (SOuRCe), Royal Prince Alfred Hospital, Missenden Road, PO Box M157, Sydney, NSW 2050, Australia

**Keywords:** Colorectal cancer, Survivorship, Models of care, Nurse-led services

## Abstract

**Background:**

Colorectal cancer (CRC) is the most common cancer affecting both men and women in Australia. The illness and related treatments can cause distressing adverse effects, impact on emotional and psychological well-being, and adversely affect social, occupational and relationship functioning for many years after the end of treatment or, in fact, lifelong. Current models of follow-up fail to address the complex needs arising after treatment completion. Strategies to better prepare and support survivors are urgently required. We previously developed a nurse-led supportive care program (SurvivorCare) and tested it in a pilot study involving 10 CRC survivors. The intervention was found to be highly acceptable, appropriate, relevant and useful.

**Methods/design:**

This study is a multisite, randomised controlled trial, designed to assess the impact of the addition of the SurvivorCare intervention to usual post-treatment care, for people with potentially cured CRC. SurvivorCare comprises the provision of survivorship educational materials, a tailored survivorship care plan, an individually tailored nurse-led, face-to-face end of treatment consultation and three subsequent telephone calls. Eligible patients have completed treatment for potentially cured CRC. Other eligibility criteria include stage I to III disease, age greater than 18 years and adequate understanding of English. All consenting patients complete questionnaires at three time points over a six-month period (baseline, two and six months). Measures assess psychological distress, unmet needs and quality of life*.*

**Discussion:**

This supportive care package has the potential to significantly reduce individual suffering, whilst reducing the burden of follow-up on acute cancer services through enhanced engagement with and utilisation of general practitioners and community based services. If the intervention is successful in achieving the expected health benefits, it could be disseminated readily. All training and supporting materials have been developed and standardised. Furthermore, the intervention could easily be adapted to other cancer or chronic disease settings.

**Trial registration:**

Australian New Zealand Clinical Trial Registry ACTRN12610000207011.

## Background

In Australia, bowel cancer is the most common cancer affecting both men and women [1]. Although it is the second highest cause of death from cancer (after lung cancer), many people are long-term survivors. Survivors of bowel cancer represent the third largest group of long-term cancer survivors in the Western world (after survivors of breast and prostate cancer) with five-year survival rates of approximately 55% [2]. It is expected that the number of people affected by and surviving bowel cancer will rise significantly over the next 10 years. Australian data showed a 30% increase in the number of new or surviving cases of colorectal cancer (CRC) in women and 33% for men between 2001 and 2011 [1].

CRC survivors are vulnerable to a number of distressing long-term sequelae [3-8]. Forty percent of people thought to be cured will ultimately develop recurrent disease [9]. In addition, survivors of CRC are at increased risk of developing a second cancer, either bowel cancer or another primary cancer [9]. Survivors are susceptible to surgical complications, such as bowel obstruction, abdominal wall hernias and altered bowel function [4,5].

Rectal cancer survivors are at risk of faecal, bladder and sexual dysfunction [5-8]. Bowel dysfunction may include faecal frequency and urgency, faecal leakage, and the need to use pads. Surgery and radiation treatment for rectal cancer may result in long-term and late consequences, such as chronic diarrhoea, poor food absorption, potential early menopause in some women, loss of fertility in both men and women, damage to the vagina and impaired ability to achieve erections [6-9]. Chemotherapy can result in persistent peripheral neuropathy resulting in pain and loss of function, impacting upon activities of daily life.

Given these serious side-effects, not surprisingly social, occupational and relationship functioning commonly suffer [9-13]. Many survivors do not return to full-time work, or may have a delayed return to work [14]. This, in addition to out of pocket expenses, can impose a significant financial burden [10,11]. Some survivors experience reduced social integration and changes in their intimate relationships. Many suffer ongoing psychosocial distress, especially those managing permanent colostomies [7,8,10]. Studies suggest an increased rate of depression compared with matched groups unaffected by cancer [12]. Schag and colleagues found, even at five-year follow-up, that many colon cancer survivors reported difficulties with physical functioning, poor energy levels, difficulty undertaking recreational activities, ongoing concerns about recurrence, body image concerns, problems with sexual interest and functioning, and work problems [13]. Despite these significant challenges, colorectal cancer survivors are underrepresented in survivorship research in general [15]. The current investigators were unable to identify any published research describing interventions to better support bowel cancer survivors following treatment completion.

The current project seeks to evaluate an innovative nurse-led supportive care program (SurvivorCare). The intervention was developed by experts and key stakeholders in supportive care research after extensive quantitative and qualitative research with colorectal cancer survivors (consumers) and health care professionals [16,17]. Consumer and health care professionals’ perspectives were sought to determine the relevance of the proposed Survivorship Care Plan (SCP) elements [16]. Specifically, the intervention is designed to: 1) be patient-centred by eliciting individual concerns and needs to direct personal health care plans; 2) provide comprehensive post-treatment care coordination by a specialist intervention nurse to address the multifaceted supportive care post-treatment needs of this patient group; 3) provide instruction in evidence-based self-care and stress reduction strategies to better enable people to manage self-care requirements in the period of cancer survivorship following treatment completion; 4) provide tailored information to patients and their significant others in order to maximise information relevance to this specific survivorship period; 5) if not already implemented, coordinate appropriate post-treatment care referrals to community and multidisciplinary health professionals where relevant; and 6) provide a sustainable model of nurse-led survivorship care centred on standardised end of treatment processes and enhanced collaboration and communication with primary care.

The complete SurvivorCare package (DVD, booklet, Question Prompt List (QPL), Survivorship Care Plan (SCP), end of treatment session and telephone follow-up) was trialed with 10 participants to examine appropriateness, acceptability and clinical feasibility in a pilot test trial. SurvivorCare proved to be a well-received, comprehensive intervention by the participants and nurses [18].

### Study aim

This study seeks to examine the effectiveness of an innovative supportive care program (SurvivorCare) comprising survivorship educational materials, provision of a tailored SCP, an individually tailored nurse-led end of treatment consultation and telephone follow-up for people with potentially cured colorectal cancer, aiming to reduce psychological distress and unmet needs in the six months following treatment completion.

### Study hypotheses

#### Primary endpoint

CRC survivors allocated to the SurvivorCare intervention will report a significant relative benefit for psychological distress compared to survivors in the control group from baseline to Follow-up 1 (eight weeks after randomisation for the usual care group, and eight weeks after the first intervention session for the SurvivorCare group).

#### Secondary endpoints

Compared to patients in control groups, patients allocated to SurvivorCare will report:

(i) Significant beneficial effect for unmet psychological and informational needs, and quality of life from Baseline to Follow-up 1;

(ii) Sustained improvement in psychosocial outcomes over six months.

## Methods

### Design and setting

The study is being undertaken at 18 sites within Australia. Peter MacCallum Cancer Centre is the coordinating site, with all participant data stored centrally. A feasibility survey was conducted in January 2009. Sites were selected based on their proximity to patients in addition to the availability of at least one nurse to administer the SurvivorCare intervention. Ideally, the nurse would be a specialist colorectal cancer nurse; however, nurses with general oncology experience were also eligible. Availability of site resources/space to conduct the study was also considered.

Ethical approval was obtained individually by the Human Research Ethics Committees of Victorian and Tasmanian sites, and a central governance approval was obtained for all sites in New South Wales.

### Participants

To be eligible, participants need to: (1) have a confirmed diagnosis of colon or rectal cancer; (2) have stage I to III disease (that is, non-metastatic); (3) be treated with curative intent with surgery +/− radiation +/− chemotherapy; (4) be over 18 years; and (5) be able to understand English. Exclusion criteria are (1) demonstrated cognitive or psychological difficulties as defined by the treatment team’s assessment or documented within the patient’s medical history; (2) too unwell to participate in the study as determined by the patient’s treatment team; (3) past history of malignancy other than non-melanomatous skin cancer; (4) enrolment in a conflicting supportive care trial that might impact the study’s measured endpoints.

### Recruitment process

**Figure 1:**
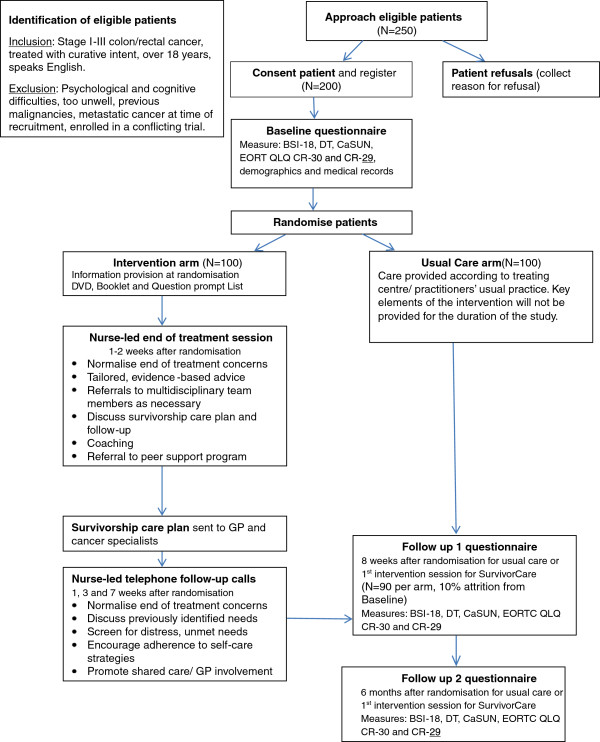
Schematic diagram of Survivor Care study design.

Patient eligibility is confirmed with the treating clinician. The study is further explained to patients by a trained data manager and written consent is obtained. Patients are approached zero to six months prior to the end of treatment (preferably), or up to six months after the end of treatment. Once the study coordinating centre receives a patient randomisation request by fax or email from a participating site, the patient is randomised 1:1 to either usual care or SurvivorCare (100 patients are expected in each arm). The type of treatment and hospital site is entered in an Access database, where the allocation is balanced by site using a minimisation method. Figure 1 illustrates a schematic diagram of the study design.

### Intervention group/SurvivorCare

The intervention consists of four main components: (1) information package; (2) nurse-led face-to-face end of treatment session; (3) SCP; and (4) telephone follow-up.

(1) Information package

The package includes a DVD (Just Take It Day to Day) [19], a booklet (*Life After Cancer*) [20], and a Question Prompt List (QPL). The QPL is a list of 70 questions covering common issues cancer survivors encounter in the first year following treatment. Ten main topics covering issues and concerns around coping, physical consequences of completing treatment, late and long-term effects of treatment, impact on family and friends, recurrence, follow-up planning, fertility, pregnancy and menopause, sexuality, dealing with practical matters, such as finances, returning to work and insurance, and need for further information and support, are listed to help the patient think about what they might like to ask their nurse or doctor. The patient is asked to view the material and complete the QPL prior to the nurse-led face-to-face session.

(2) Nurse-led face-to-face session

A nurse-led end of treatment session will take place in a standard timeframe after randomisation. Focus group results and a review of the literature [17] and expert input were used to design the end of treatment session, which addresses current concerns, seeks to attain a sense of closure on treatment and aims to prepare people for the survivorship phase and introduce telephone follow-up. The session takes place in a private hospital clinic room by a nurse trained to deliver the intervention. Session time is 60 to 90 minutes. Significant others are encouraged to also attend the end of treatment consultation to maximise the positive impact on patient outcomes [21,22]. Additional concerns not covered in the session are covered in subsequent telephone follow-up sessions. The session incorporates seven elements.

### Normalising treatment completion concerns

The vast majority of patients feel a degree of uncertainty for what lies ahead. Discussing treatment completion and the future will be addressed for all patients in the intervention arm.

### Tailored, evidence-based information and advice

At the beginning of the session, survivors score their current level of distress using the Distress Thermometer (DT) and indicate any queries or questions using the QPL. Level of distress noted on the DT and problem areas identified through the QPL will be used to guide a discussion of individual needs and prompt referral to other services. The patient will be asked to identify their top three concerns for focus during the session. Evidence-based self-management strategies will be provided to deal with side-effects. An intervention manual includes evidence-based guidance regarding all 69 questions from the QPL; together with additional information and resources (for example, information and support services for people with a stoma or managing continence issues). All information, processes and outcomes from the session are recorded in an End of Treatment Session Checklist.

### Referral to multi-disciplinary and community referrals

Survivors may experience a range of unmet supportive care needs. Research shows that nurse co-ordinated multi-disciplinary and community referrals result in better symptom control for cancer patients [23]. Nurses will offer (and organise where necessary) appropriate multidisciplinary and community referrals for unmet needs identified on the problem checklist from the DT or QPL.

### Recommendations regarding follow-up and discussion of the survivorship care plan

The SCP includes details of the cancer diagnosis, treatments, health promotional advice, supportive care and psychosocial elements, as well as recommendations for follow-up to detect cancer recurrence. The SCP will be reviewed and the importance of follow-up to screen for physical and supportive care issues will be emphasised. Patients will be given a SCP during the end of treatment session. Their general practitioner and oncology specialists will also be sent a copy.

### Liaison with the patient’s GP

Patients will be encouraged to discuss aspects of their survivorship care that might be best transitioned to their GP. Discussion will also cover aspects of care being managed by the patient’s GP and aspects of care for management by the specialist. This will be documented in the SCP. Patients will be advised that their GPs will also receive the SCP and recommendations regarding detection of cancer recurrence and screening for survivorship needs. The benefits of shared care with the GP will be emphasised. Thus, the transition from intensive hospital treatment to community-based GP maintenance, mediated through nurse-led phone follow-up will be encouraged. Discussion will also include appropriate time points for follow-up by medical specialists.

### Coaching

Health promotional strategies, such as stopping smoking, exercising and losing weight will be discussed where these are identified as issues for the survivor or the health professional. Intervention nurses will be trained to assess patients’ readiness to make behavioural change and will assist participants to establish behavioural change goals. Coaching and rehearsal of self-care for side-effects and stress-reduction strategies will be offered and reinforced during telephone follow-up sessions.

### Referral to peer support programs

All participants will be referred to peer support programs. Cancer Councils operate a free, one-to-one telephone-based peer support program called Cancer Connect. Volunteers are CRC survivors who have received rigorous training, including communication skills (asking open questions, empathy, active listening) and encouraging coping and adherence to self-care and health promotional advice. Patients will also be advised of other community-based resources, including cancer support groups and the Cancer Council Helpline.

### Nurse-led telephone follow-up

These sessions will take place one, three and seven weeks after the first intervention session. The purpose of the sessions are to: a) support the patient during the post treatment completion period to reduce feelings of isolation or abandonment from the hospital team; (b) revisit needs discussed during the end of treatment session and explore in detail and respond to any concerns not addressed at that session; (c) screen for psychosocial distress and additional unmet needs using the Distress Thermometer, issues highlighted in the QPL and the Survivor Care Plan; (d) encourage adherence to recommended self-care strategies and planned health behaviour change goals, and (e) emphasise the collaborative relationship between the patient’s GP and hospital team, highlighting that the patient’s GP has been provided with relevant information to enable effective shared care. Appointments will be made for the three calls, at the end of treatment session. The patient’s shared care team will be notified of any new concerns identified by the patient or nurse during telephone follow-up, as well as significantly high levels of psychosocial distress, and additional unmet needs. New patient concerns will be followed up by the intervention nurse at subsequent telephone follow-ups. Table 1 offers a summary of the elements covered by the intervention during the initial and follow-up sessions.

**Table 1:** Features of the intervention covered during the post end of treatment sessions

**Elements of the SurvivorCare**	**Covered**	**Covered during**
**intervention**	**during initial**	**follow-up**
	**session**	**sessions**
Discussion of the survivorship care plan and introduction to follow-up	Yes	
Coaching	Yes	
Referral to peer support programs	Yes	
Normalising of common end of treatment concerns	Yes	
Discuss current concerns and advice	Yes	Yes
Referral to multi-disciplinary and community referrals	Yes	Yes
Screen for distress	Yes	Yes
Encourage adherence to self-care strategies	Yes	Yes
Promote shared care/GP involvement	Yes	Yes

### Usual care/control group

Participants allocated to usual care will receive care according to the treating centre/practitioner’s usual practice. Key elements of the intervention will not be provided in the control group (tailored, nurse-led, face-to-face and telephone sessions, question prompt list, self-care resources, SCP and GP correspondence). Participating health care services will be asked not to incorporate similar elements into usual care for the duration of the study. Participants allocated to the usual care group will be asked whether they have received any components of the SurvivorCare intervention, for example, information materials, SCP.

### Intervention staff training

Intervention nurses with experience looking after people with bowel cancer have been trained to provide the end of treatment and telephone follow-up sessions at each site. An expert in communication skills training and two experts in nurse-led clinics and self-care education conducted a full-day training program comprising: (a) an overview of the project, emphasising prevention of diffusion of the intervention at the site; (b) explanation of how to use the intervention manual; (c) training in eliciting and responding to emotional cues, including dealing with distress; (d) instruction on exploring concerns and providing evidence-based advice; (e) instruction in how to effectively facilitate referral to multidisciplinary team members; (f) instruction on how to coach patients to use evidence-based strategies; (g) role-play with simulated patients (actors) and activity sessions to practice the intervention, including self-appraisal and constructive feedback.

### Data manager training

Data managers (DMs) with experience in participant recruitment and coordinating research projects were trained to identify eligible patients, liaise with hospital staff and intervention nurses, approach patients, administer surveys, manage participant databases, and report data back to the project team. DMs were also trained on patient randomisation techniques.

### Study integrity and quality assurance

Efforts will be made to ensure that the intervention nurse will not be involved in follow-up of patients receiving usual care, to avoid contamination. At sites where only one nurse is available to administer both the intervention and usual care to patients, the intervention nurse will be given specific training to ensure that patients in the control group will not be given additional post-treatment care in addition to usual care.

A usual care checklist will be sent out with the first follow-up questionnaire to all patients in the usual care arm to determine whether patients have received elements of the intervention, such as the DVD, the booklet or SCP.

An intervention manual summarising details of the intervention was developed to train the nurses delivering the intervention. Intervention procedures will strictly be monitored for adherence to the manual. All intervention sessions will be audio-taped. The first 10 sessions will be reviewed to ensure adherence against a checklist derived from the manual. After this, a random sample of 15% of all sessions will continue to be reviewed. To ensure that competency is maintained, regular feedback sessions will be arranged.

Reasons for attrition will be recorded, and recruitment and drop-out bias assessed.

### Data collection procedure

Participants will be asked to complete a baseline questionnaire on the day of randomisation and two subsequent follow-up questionnaires at eight weeks (Follow-up 1) and six months (Follow-up 2) after the face-to-face session for the intervention group or after randomisation for the usual care group. Table 2 shows data to be collected.

**Table 2:** Time points measuring patient reported outcomes for the SurvivorCare study

**Time point**	**Questionnaire**
Baseline: on the day of randomisation (zero to two weeks before a potential first intervention session)	Demographic variables, Medical records, BSI-18, DT, CaSUN, EORTC QLQ C-30 and CR-29.
Follow-up 1: eight weeks after randomisation date for usual care patient or after the first session date for intervention patients	BSI-18, DT, CaSUN, EORTC QLQ C-30 and CR-29 and Satisfaction Measure with usual care checklist
Follow-up 2: six months after randomisation date for usual care patient or the first session date for intervention patients	BSI-18, DT, CaSUN, EORTC QLQ C30 and CR-29

BSI-18, Brief Symptom Inventory; CaSUN, Cancer Survivors’ Unmet Needs Measure; DT, Distress Thermometer; EORTC QLQ C-30, European Organisation for Research and Treatment of Cancer core questionnaire; EORTC QLQ CR-29, the colorectal cancer module.

### Demographic information

Data to be collected include age, gender, marital status, age of any children, postcode, occupation and education level.

### Medical records

Data will include cancer type, stage, treatment, time since diagnosis, treatment outcomes, recurrence, treatment side-effects/toxicities, performance status, other non-cancer morbidities, unscheduled/emergency appointments at treating centre and other identified centres, admissions as an inpatient, addition to or changes in medication prescriptions to manage toxicities and referrals to allied health services at the treating centre.

### Psychological distress

The *Brief Symptom Inventory 18 (BSI-18)* was selected as the primary outcome measure [24]. It comprises 18 items answered on a 5-point scale to score global distress. Subscales assess anxiety, depression and somatisation. It has demonstrated reliability, validity, acceptability and responsiveness and has been used extensively in both cancer and general populations. It has been recommended for use in identifying psychological distress in a clinical setting [25]. The *Distress Thermometer (DT)*[26] is a reliable and valid single item measure of distress in cancer populations, which appears responsive to change [27].

### Survivors unmet needs

*Cancer Survivors’ Unmet Needs measure (CaSUN)*[28] will be used to assess survivors’ unmet needs. It includes 35 unmet need items, 6 positive change items and an open-ended question. The scale has good acceptability, internal consistency and validity. A review of measures of survivors’ unmet needs suggests that the CaSUN performs as well, or better, than any other measure [29].

### Quality of life

The European Organisation for Research and Treatment of Cancer core questionnaire (EORTC QLQ C-30) [30] will be used together with the colorectal cancer module (EORTC QLQ CR-29) [31] to measure quality of life. Together, the scales include 59 items that assess quality of life issues specific to this patient group. The measures are widely used, with good test-retest reliability, validity, responsiveness and acceptability.

### Sample size requirements

The primary outcome for this longitudinal randomised controlled trial is psychological distress as assessed by the BSI-18 Global Severity Index, an 18-item scale with a possible range of 72. The BSI-18 will be completed once at baseline and twice post-intervention, but the main contrast of interest is the difference between arms at the first post-intervention follow-up.

In this case, we based our sample size calculations on 80% power, a two-sided *t*-test with an alpha-level of 0.05, a difference between groups of 3.6 points on the primary outcome (or 5% of the instrument range) [32] and a standard deviation of 8.6 [24] (a standardised difference of 0.42). Given these specifications, the required sample size is 180 patients (or 90 patients per arm). Assuming attrition of up to 10%, 200 patients will need to be recruited (or 100 patients per arm). Furthermore, assuming an 80% recruitment rate, approximately 250 patients will need to be approached to achieve a total sample of 200 patients. Patients will be recruited over 24 months.

### Statistical analysis

Analyses will be by intention-to-treat and performed in SPSS Windows Version 21.0 (or higher) (Chicago, IL, USA). Prior to formal analyses, descriptive statistics and graphical displays will be used to identify missing and out-of-range values, assess the distributional characteristics of test scores and summarise study measure completion rates and missing data points. Responses to the BSI-18, DT, CaSUN, EORTC QLQ C-30 and CR-29 will be scored according to algorithms in the users’ manuals for these instruments.

Descriptive statistics will be used to summarise baseline demographic and clinical characteristics of patients randomised to the intervention and usual care. Recruitment bias and possible differential attrition will be assessed by comparing basic demographic and clinical characteristics of responders and non-responders, as well as drop-outs and continuing participants, using *t*-tests (or Mann–Whitney U) and chi-squared tests as appropriate.

Primary and secondary outcome analyses will be carried out by fitting a linear mixed model to each outcome separately using all available data. A cell means model will be used to estimate mean scores for each group by time combination. An unstructured covariance type will be used to model the covariance structure among repeated measures and all models will be estimated by maximum likelihood. Group comparisons at post-baseline time points will be performed using contrasts within the proposed models. After inspection of the data, the appropriateness of stated methods will be revised, if necessary. Because of the number of significance tests for secondary outcomes, Hochberg’s modified Bonferroni procedure will be used so that the overall type I error rate does not exceed 0.05 [33].

## Discussion

CRC survivors represent a large and rapidly growing group of people. It is critical that their serious and often distressing needs following treatment completion are appropriately managed. Unfortunately, there are few interventional studies that seek to improve psychosocial and supportive care outcomes for cancer survivors and the investigators identified no studies for patients with CRC, following treatment completion.

This study will examine the impact of a nurse-led intervention package for CRC survivors using important clinical outcomes, such as distress, anxiety and depression, as well as unmet needs and quality of life. SurvivorCare recognises treatment completion as a critical period. The intervention is patient-centred, individually tailored and nurse-led with active GP engagement. It screens for distress, provides evidence based information and coaching in self-care and stress reduction. Patients are offered health promotional strategies and, where appropriate, referred to a broader multidisciplinary team. The effectiveness of the intervention will be assessed in terms of important clinical outcomes, specifically distress, anxiety and depression, unmet needs and quality of life.

SurvivorCare has the potential to considerably reduce individual suffering, but it is also likely to reduce the cost of health provision to this group of patients, as failing to meet patients’ psychological and supportive care needs results in longer hospital stays and higher medical costs [34]. If the intervention is successful in achieving the expected health benefits, it could be disseminated readily. Furthermore, the intervention could easily be adapted to other cancer or chronic disease settings. By encouraging consumer involvement in health care planning and promoting shared care between specialist treatment centres and community services, the proposed research program has the potential to transform health care practices.

## Trial status

Patient recruitment is open.

## Abbreviations

CRC: Colorectal cancer; DMs: Data managers; DT: Distress thermometer; QPL: Question prompt list; SCP: Survivorship care plan.

## Competing interests

The authors declare that they have no competing interests.

## Authors’ contributions

MJ conceptualised and designed the study, and led the development of this paper. He participated in the implementation of the protocol and provided health professional training. SA assisted in the development of the protocol, the study design and the refinement of study materials. KG assisted in the study design with sample size calculations and statistical analysis. KLJ, PB, MK, JY and DK assisted in the development of the protocol, the study design and the refinement of study materials. JPN participated in the implementation of the protocol and data collection. LR participated in the implementation of the protocol, coordinated the data collection, provided data manager training and led the development of this paper. PS assisted in the development of the protocol, the study design and the refinement of study materials, and provided health professional training. All authors have been involved in drafting and critical evaluation of the manuscript. All authors have read and approved the final version.

## References

[B1] McDermid I AIHW, AACR, NCSG Cancer Incidence Projections Australia 2002 to 2011 2005 Canberra, ACT: Australian Institute of Health and Welfare (AIHW), Australasian Association of Cancer Registries (AACR) and the National Cancer Strategies Group (NCSG)

[B2] Jemal A Bray F Center MM Ferlay J Ward E Forman D Global cancer statistics CA Cancer J Clin 2011 61 69 90 10.3322/caac.20107 21296855

[B3] Edna T-H Bjerkeset T Small bowel obstruction in patients previously operated on for colorectal cancer Eur J Surg 1998 164 587 592 9720935 10.1080/110241598750005688

[B4] Cahalane M Shapiro M Silen W Abdominal incision: decision or indecision? Lancet 1989 333 146 148 10.1016/S0140-6736(89)91154-9 2563057

[B5] Camilleri-Brennan J Steele RJ Prospective analysis of quality of life and survival following mesorectal excision for rectal cancer Br J Surg 2001 88 1617 1622 10.1046/j.0007-1323.2001.01933.x 11736975

[B6] Moriya Y Function preservation in rectal cancer surgery Int J Clin Oncol 2006 11 339 343 10.1007/s10147-006-0608-z 17058130

[B7] Nugent K Daniels P Stewart B Patankar R Johnson C Quality of life in stoma patients Dis Colon Rectum 1999 42 1569 1574 10.1007/BF02236209 10613475

[B8] Sprangers MAG Taal BG Aaronson NK Velde A Quality of life in colorectal cancer Dis Colon Rectum 1995 38 361 369 10.1007/BF02054222 7720441

[B9] Goldberg RM Fleming TR Tangen CM Moertel CG Macdonald JS Haller DG Laurie JA Surgery for recurrent colon cancer: strategies for identifying resectable recurrence and success rates after resection Ann Intern Med 1998 129 27 35 10.7326/0003-4819-129-1-199807010-00007 9652996

[B10] Arndt V Merx H Stegmaier C Ziegler H Brenner H Restrictions in quality of life in colorectal cancer patients over three years after diagnosis: a population based study Eur J Cancer 2006 42 1848 1857 10.1016/j.ejca.2006.01.059 16829069

[B11] Institute of Medicine, National Research Council Hewitt M, Greenfield S, Stovall E From Cancer Patient to Cancer Survivor: Lost in Transition. An American Society of Clinical Oncology and Institute of Medicine Symposium 2006 Washington, DC: National Academies Press

[B12] Ramsey SD Berry K Moinpour C Giedzinska A Andersen MR Quality of life in long term survivors of colorectal cancer Am J Gastroenterol 2002 97 1228 1234 10.1111/j.1572-0241.2002.05694.x 12017152

[B13] Schag CA Ganz PA Wing DS Sim MS Lee JJ Quality of life in adult survivors of lung, colon and prostate cancer Qual Life Res 1994 3 127 141 10.1007/BF00435256 8044158

[B14] Short PF Vasey JJ Tunceli K Employment pathways in a large cohort of adult cancer survivors Cancer 2005 103 1292 1301 10.1002/cncr.20912 15700265

[B15] Aziz N Rowland J Cancer survivorship research among ethnic minority and medically underserved groups Oncol Nurs Forum 2002 29 789 801 10.1188/02.ONF.789-801 12058154

[B16] Baravelli C Krishnasamy M Pezaro C Schofield P Lotfi-Jam K Rogers M Milne D Aranda S King D Shaw B Grogan S Jefford M The views of bowel cancer survivors and health care professionals regarding survivorship care plans and post treatment follow up J Cancer Surviv 2009 3 99 108 10.1007/s11764-009-0086-1 19415504

[B17] Jefford M Karahalios E Pollard A Baravelli C Carey M Franklin J Aranda S Schofield P Survivorship issues following treatment completion-results from focus groups with Australian cancer survivors and health professionals Journal of Cancer Survivorship 2008 2 20 32 10.1007/s11764-008-0043-4 18648984

[B18] Jefford M Lotfi-Jam K Baravelli C Grogan S Rogers M Krishnasamy M Pezaro C Milne D Aranda S King D Shaw B Schofield P Development and pilot testing of a nurse-led posttreatment support package for bowel cancer survivors Cancer Nurs 2011 34 E1 E10 21045754 10.1097/NCC.0b013e3181f22f02

[B19] Karahalios A Baravelli C Carey M Schofield P Pollard A Aranda S Franklin J Jefford M An audiovisual information resource to assist in the transition from completion of potentially curative treatment for cancer through to survivorship: A systematic development process Journal of Cancer Survivorship 2007 1 226 236 10.1007/s11764-007-0022-1 18648973

[B20] Jefford M Karahalios A Angle A Baravelli C Akkerman D Understanding issues for cancer survivors: informing the development and evaluation of a written information booklet for survivors at treatment completion Psychooncology 2007 16 S273

[B21] Barlow J Wright C Sheasby J Turner A Hainsworth J Self-management approaches for people with chronic conditions: a review Patient Educ Couns 2002 48 177 187 10.1016/S0738-3991(02)00032-0 12401421

[B22] Deadman JM Leinster SJ Owens RG Dewey ME Slade PD Taking responsibility for cancer treatment Soc Sci Med 2001 53 669 677 10.1016/S0277-9536(00)00369-5 11478545

[B23] Addington-Hall JM MacDonald LD Anderson HR Chamberlain J Freeling P Bland JM Raftery J Randomised controlled trial of effects of coordinating care for terminally ill cancer patients BMJ 1992 305 1317 1322 10.1136/bmj.305.6865.1317 1483075 PMC1883850

[B24] Zabora J Brintzenhofeszoc K Jacobsen P Curbow B Piantadosi S Hooker C Owens A Derogatis L A new psychosocial screening instrument for use with cancer patients Psychosomatics 2001 42 241 246 10.1176/appi.psy.42.3.241 11351113

[B25] Love A; National Breast Cancer Centre The Identification of Psychological Distress in Women with Breast Cancer 2004 Camperdown, NSW, Australia: National Breast Cancer Centre

[B26] Jacobsen PB Donovan KA Trask PC Fleishman SB Zabora J Baker F Holland JC Screening for psychologic distress in ambulatory cancer patients Cancer 2005 103 1494 1502 10.1002/cncr.20940 15726544

[B27] Gessler S Low J Daniells E Williams R Brough V Tookman A Jones L Screening for distress in cancer patients: is the distress thermometer a valid measure in the UK and does it measure change over time? A prospective validation study Psychooncology 2008 17 538 547 10.1002/pon.1273 17973237

[B28] Hodgkinson K Butow P Hunt GE Pendlebury S Hobbs KM Lo SK Wain G The development and evaluation of a measure to assess cancer survivors’ unmet supportive care needs: the CaSUN (cancer survivors’ unmet needs measure) Psychooncology 2007 16 796 804 10.1002/pon.1137 17177268

[B29] Pearce NJ Sanson-Fisher R Campbell HS Measuring quality of life in cancer survivors: a methodological review of existing scales Psychooncology 2008 17 629 640 10.1002/pon.1281 17973235

[B30] Aaronson NK Ahmedzai S Bergman B Bullinger M Cull A Duez NJ Filiberti A Flechtner H Fleishman SB de Haes JC Kaasa S Klee M Osoba D Razavi D Rofe PB Schraub S Sneeuw K Sullivan M Takeda F The European Organization for Research and Treatment of Cancer QLQ-C30: a quality-of-life instrument for use in international clinical trials in oncology J Natl Cancer Inst 1993 85 365 376 10.1093/jnci/85.5.365 8433390

[B31] Sprangers MA te Velde A Aaronson NK The construction and testing of the EORTC colorectal cancer-specific quality of life questionnaire module (QLQ-CR38) Eur J Cancer 1999 35 238 247 10.1016/S0959-8049(98)00357-8 10448266

[B32] King MT A point of minimal important difference (MID): a critique of terminology and methods Expert review of pharmacoeconomics & outcomes research 2011 11 171 184 10.1586/erp.11.9 21476819

[B33] Hochberg Y A sharper Bonferroni procedure for multiple tests of significance Biometrika 1988 75 800 802 10.1093/biomet/75.4.800

[B34] Himelhoch S Weller WE Wu AW Anderson GF Cooper LA Chronic medical illness, depression, and use of acute medical services among medicare beneficiaries Med Care 2004 42 512 521 10.1097/01.mlr.0000127998.89246.ef 15167319

